# Characterization of gut-homing molecules in non-endstage livers of patients with primary sclerosing cholangitis and inflammatory bowel disease

**DOI:** 10.1016/j.jtauto.2020.100054

**Published:** 2020-04-09

**Authors:** Manon de Krijger, Thijmen Visseren, Manon E. Wildenberg, Gerrit K.J. Hooijer, Monique M.A. Verstegen, Luc J.W. van der Laan, Wouter J. de Jonge, Joanne Verheij, Cyriel Y. Ponsioen

**Affiliations:** a Tytgat Institute for Liver and Intestinal Research, Amsterdam UMC, University of Amsterdam, Meibergdreef 69, 1105 BK, Amsterdam, the Netherlands; b Department of Gastroenterology and Hepatology Amsterdam UMC, University of Amsterdam, Meibergdreef 9, 1105 AZ, Amsterdam, the Netherlands; c Department of Gastroenterology and Hepatology, Erasmus MC-University Medical Center Rotterdam, Doctor Molewaterplein 40, 3015 GD, Rotterdam, the Netherlands; d Department of Pathology, Amsterdam UMC, University of Amsterdam, Meibergdreef 9, 1105 AZ, Amsterdam, the Netherlands; e Department of Surgery, Erasmus MC-University Medical Center Rotterdam, Doctor Molewaterplein 40, 3015 GD, Rotterdam, the Netherlands

**Keywords:** Primary sclerosing cholangitis, Lymphocyte recruitment, Gut-homing, Immune-mediated liver disease

## Abstract

**Introduction:**

The co-occurrence of inflammatory bowel disease (IBD) in up to 80% of patients with primary sclerosing cholangitis (PSC) suggests a relation between the gut and the liver in patients with both PSC and IBD. One hypothesis suggests that aberrantly expressed homing molecules in the liver drive infiltration of gut-homing memory T-cells that are originally primed in intestinal environment. One of the main findings supporting this hypothesis is the expression of mucosal addressin cell adhesion molecule 1 (MAdCAM-1) in PSC livers. Expression of homing molecules in early PSC remains unclear. The aim of this study was to investigate expression patterns of homing chemokines and adhesion molecules in PSC-IBD colons and livers, and to study whether changes are already present in early stages of PSC.

**Methods:**

Needle biopsies from livers of 20 PSC patients with short-term PSC (PSC-IBD_ST_) as well as explant liver biopsies of 8 patients with long-term PSC (PSC-IBD_LT_) were collected (median disease duration 0 and 22 years, respectively). Only patients with concomitant IBD were included (89% ulcerative colitis and 11% Crohn’s disease). Expression and distribution of MAdCAM-1, VAP-1, integrin β7, CCL25, CCL28, CXCL12, αE (CD103) and E-cadherin were assessed in both liver and colon tissue. Liver tissue collected from obstructive cholangitis in resection specimens for Klatskin tumors or resection specimens from hepatic metastasis, liver tissue of patients with hepatitis C virus (HCV) and of patients with primary biliary cholangitis (PBC) served as controls.

**Results:**

MAdCAM-1 expression in livers of PSC-IBD_LT_ patients was increased compared to controls. The proportion of CD3^+^ T-cells expressing integrin β7 did not differ between PSC-IBD_ST_ and control groups, but was higher in liver tissue of PSC-IBD_LT_ patients. There was no difference in αE^+^ T-cells between PSC-IBD_LT_ and control groups. The chemokine CCL28 was highly expressed in biliary epithelial cells. This intense staining pattern was more pronounced in PSC-IBD_ST_, but overall did not significantly differ from controls.

**Conclusions:**

We confirm that aberrant gut lymphocyte homing to the liver exists in PSC, linking gut and liver disease pathology in PSC-IBD. Our data suggests that this phenomenon increases over time in later stages of the disease, worsening ongoing inflammation.

## Highlights

• MAdCAM-1 is aberrantly expressed in PSC-IBD liver compared to control liver.
• The proportion of beta7 positive T-cells is increased in long-term PSC livers.
• Biliary epithelial cells express CCL28, already in short-term PSC.
• Expression patterns of inflammatory cytokines in PSC-IBD colon are not distinct from IBD.

## 1 Introduction

Primary sclerosing cholangitis (PSC) is an uncommon chronic inflammatory disease of the liver. In patients with PSC, inflammation of the intra- and extrahepatic bile ducts leads to strictures and fibrosis, ultimately resulting in cirrhosis and end-stage liver disease. It is a grave disease with a median liver transplant-free survival of 21 years [1]. PSC is strongly associated with inflammatory bowel diseases (IBD), which occurs in approximately 70% of all PSC patients. The majority of these patients has ulcerative colitis [1,2]. In addition, it has been described that histopathological signs of inflammation are present in a subset of patients that have no endoscopic signs of inflammation, suggesting that this co-occurrence is even higher [3].

The co-occurrence of PSC and IBD has fueled the hypothesis of an interaction between the gut and the liver, known as ‘the aberrant gut homing lymphocyte paradigm’ [4]. This hypothesis describes an abnormal expression of homing molecules in PSC liver, leading to unusual aggregation of gut-primed memory T-cells into the liver. The recruitment of lymphocytes from the circulation to mucosal tissues such as the gut is a complex process involving a wide range of chemokines and contributing adhesion molecules. After recognition of an antigen in the gut, dendritic cells can prime naïve T-cells with gut-specific receptors: integrin α4β7 and CC-chemokine receptor 9 (CCR9) [5]. This will allow these gut-primed T-cells to home back to the intestine via Mucosal Addressin Cell Adhesion Molecule-1 (MAdCAM-1), which is constitutively expressed on venular endothelium of the gut lamina propria and high endothelial venules in Peyer’s patches and mesenteric lymph nodes [6]. An interaction between α4β7 with MAdCAM-1 promoted by CCR9 and CCL25 will facilitate lymphocyte adhesion to endothelium [7,8]. Although CCR9 is widely expressed on lymphocytes in the small intestine, in the large intestine only a small number of lymphocytes express CCR9 [9,10]. In the colon, chemokine CCL28 is upregulated in the epithelium during inflammation, attracting T- and B-cells expressing its receptor CCR10 [11]. In flow-based adhesion assays CCL28 is able to trigger α4β7-dependent lymphocyte arrest on MAdCAM-1, in addition to CCL25 [7].

Although integrin α4β7 and CCR9 are originally described as key homing molecules to gut associated lymphoid tissue (GALT) [9], several studies have shown evidence of α4β7^+^ and CCR9^+^ lymphocytes binding to liver endothelium in PSC and presence of these cells in the peribiliary infiltrates in PSC liver [12,13]. In addition, MAdCAM-1 is upregulated in different inflammatory disorders in both gut and liver, including IBD but also PSC, primary biliary cholangitis (PBC), and hepatitis C virus (HCV) [6,[13], [14], [15]]. Additionally, adhesion molecule vascular adhesion protein (VAP)-1 is more abundant in PSC liver, which in its turn can induce MAdCAM-1 expression by endothelial cells via its enzymatic functions [16,17]. In order to be retained in the liver, lymphocytes can be attracted by various signals. CXCL12, also called stromal cell-derived factor 1 (SDF-1), is a chemokine constitutively expressed by biliary epithelial cells, of which the expression is enhanced in liver diseases like PSC, at least in end-stage disease [18]. *In vitro*, CXCL12 is able to attract PSC liver derived lymphocytes [8]. Expression of CCL28 by biliary epithelial cells is increased in inflamed human liver, and attracts a subset of CCR10^+^ lymphocytes [19]. It is hypothesized that α4β7^+^ T-cells can differentiate into αEβ7^+^ T-cells within the liver, partly regulated by TGF-β that is locally secreted by epithelial cells [20]. Although the ligand of αE (CD103), E-cadherin, seems to be less expressed in end-stage PSC liver, its expression in early PSC is unknown [21]. Since PSC is highly associated with IBD, it may be that these gut-homing mechanisms play a decisive role in the pathophysiology of PSC-IBD [22].

In the past decade, several compounds interfering with gut-homing mechanisms have been developed, for example those blocking integrin α4β7 (vedolizumab, abrilumab), integrin β7 (etrolizumab), and MAdCAM-1 (ontamalimab). Studies predominantly focus on IBD, but if these compounds are also able to block trafficking into the liver, they could also be a treatment option for PSC-IBD.

We hypothesized that adhesion molecules and chemokines normally expressed in the gut are aberrantly expressed in the liver of patients with both PSC and IBD, and that this already occurs in an early stage of the disease. However, lymphocyte trafficking has almost exclusively been studied in explant livers at the time of liver transplantation, thereby reflecting end-stage liver disease, which may merely reflect non-specific findings. In this descriptive study, we therefore characterized the different chemokines and adhesion molecules known to be involved in lymphocyte trafficking mechanisms in the gut, in livers of PSC-IBD patients at several disease phases. Hence, we focused on PSC-IBD at the time of diagnosis, as well as at end-stage disease during transplantation.

## 2 Materials and methods

### 2.1 Patients and samples

Patients diagnosed with concomitant PSC and IBD (PSC-IBD) were included. Diagnosis of PSC was established according to the guidelines of the European Association for the study of the liver, diagnosis of IBD was based on the Lennard-Jones criteria [23,24]. To investigate differences in short-term and long-term disease, patients were divided in two groups based on moment of tissue sampling. Needle biopsies from patients with PSC-IBD who underwent diagnostic liver biopsy as well as biopsies to assess disease severity at time of diagnosis were retrospectively collected, as well as tissue from patients who underwent liver surgery because of cholangiocarcinoma (18 patients and 2 patients, respectively). Disease duration in these short-term PSC-IBD patients (PSC-IBD_ST_) was less than one year (median 0 years, IQR 0–1) (Table 1). To include long-term liver disease (PSC-IBD_LT_), liver biopsies of 10 PSC-IBD patients referred for liver transplantation were collected during transplantation procedures at the Erasmus MC. Two patients with PSC/autoimmune hepatitis (AIH) overlap syndrome were excluded. Patients required transplantation because of decompensated liver cirrhosis or recurrent cholangitis. Disease duration in this long-term PSC group was 22 years (median 22 years, IQR 10–27) (Table 1). The majority of patients was diagnosed with ulcerative colitis (PSC-IBD_ST_ 100%, PSC-IBD_LT_ 63%). Age at PSC and IBD diagnosis did not differ between the two groups (Table 1). Disease stage and grade were scored according to Ludwig and Nakanuma [25,26]. Supplementary Table 1a gives an overview of the liver tissues and performed techniques.

**Table 1:** Characteristics of patients with PSC-IBD whose liver tissue was used (short-term and long-term PSC-IBD).

	Short-term PSC-IBD (n ​= ​20)	Long-term PSC-IBD (n ​= ​8)	P-value
Male [n (%)]	15 (75)	8 (100)	0.281
Age at sampling (years) [median (IQR)]	36 (24–46)	57 (49–61)	<0.001
Age at PSC diagnosis (years) [median (IQR)]	35 (23–46)	35 (26–43)	0.823
Disease duration PSC (years) [median (IQR)]	0 (0–1)	22 (10–27)	<0.001
Small duct PSC [n (%)]	4 (20)	0 (0)	0.295
IBD type [n (%)]			0.017
Ulcerative Colitis	20 (100)	5 (63)	
Crohn’s disease	0 (0)	3 (27)	
Age at IBD diagnosis (years) [median (IQR)]	27 (20–43)	35 (26–40)	0.549
Disease duration IBD (years) [median (IQR)]	6 (0–8)	22 (13–27)	<0.001
Medication use [n (%)]			
Ursodeoxycholic acid	18 (90)	8 (100)	1.000
Ludwig Stage [n (%)]			0.010
Stage 0	2 (10)	0 (0)	
Stage 1	4 (20)	0 (0)	
Stage 2	7 (35)	0 (0)	
Stage 3	6 (30)	2 (33)	
Stage 4	1 (5)	4 (67)	

PSC, Primary Sclerosing Cholangitis: IBD, Inflammatory Bowel Disease; IQR, Interquartile range. P-values were calculated using Fisher’s Exact Test for categorical variables and Mann-Whitney U statistic for numerical variables.

Liver specimens collected from either PBC patients (biopsies at time of diagnosis; n ​= ​17), HCV patients (residual tissue from liver resections; n ​= ​7) or patients who underwent a resection because of either hilar cholangiocarcinoma or liver metastasis (further referred to as ‘control liver’; n ​= ​11) were used as control groups. None of the control patients were diagnosed with concomitant IBD.

PSC and PBC patients were part of the ‘Epi PSC PBC project’, a large population-based cohort study of PSC and PBC patients in the Netherlands [1]. All liver samples were collected with informed patient consent and local ethical committee approval (METC 06–267/E), or anonymously according to the Code of conduct for responsible use. For liver tissue collected during liver transplantation, medical ethical approval was given (MEC-2014-060), and all transplant patients signed an informed consent to use their liver biopsies for research purposes.

Biopsies of the ascending colon of PSC-IBD and IBD patients were prospectively collected during diagnostic or surveillance colonoscopy. Patients had to be older than 18 years of age, and referred for screening for IBD in case of newly diagnosed PSC or surveillance colonoscopy for PSC-IBD or IBD. In total, biopsies from 18 PSC-IBD patients as well as 11 IBD patients were collected. Median disease duration of PSC was 8 years (IQR 3–16). Only patients with diagnosis UC were included, clinical characteristics are listed in Supplementary Table 2. From two patients both liver and colonic biopsies were present, in all other cases, colon and liver tissue was not matched. The accredited Medical Ethics Committee at the Amsterdam UMC, University of Amsterdam approved the protocol (MEC 09/059) and all colon samples were collected with patient consent. Supplementary Table 1b gives an overview of intestinal samples and performed techniques.

This research was carried out in accordance with The Code of Ethics of the World Medical Association (Declaration of Helsinki) for experiments involving humans.

### 2.2 Immunohistochemical staining

Fresh liver tissue and colonic biopsies were collected after surgery or during endoscopy and fixed in formalin and embedded in paraffin (FFPE) according to standard procedure. Original liver biopsies and resection specimens in FFPE were collected from the pathology department diagnostic archives of the Amsterdam UMC, location AMC. Sections of 4,5 ​μm were cut. After deparaffinization in xylene and rehydration in ethanol series, endogenous peroxidase activity was blocked using 0.3% H_2_O_2_ in 100% methanol for 20 ​min. Heat-induced antigen retrieval was performed by pressure boiling for 20 ​min at 100 ​°C in either TRIS-based antigen unmasking solution (pH 9.0, Vector Laboratories, USA) for antibodies CXCL12, VAP-1, CD103 and E-cadherin or 0.1 ​M sodium citrate buffer (pH 6.0) for MAdCAM-1 antibody staining. Afterwards, the slides were cooled for 30 ​min on ice at 4 ​°C. Nonspecific binding sites were blocked with PBT (Phosphate Buffered Saline (PBS), Bovine Serum Albumin (BSA), Triton-X-100) for 10 ​min, after which diluted primary antibody was added and incubated overnight at 4 ​°C. Sections were incubated with either mouse anti-human MAdCAM-1 (clone 355G8, Invitrogen), mouse anti-human CXCL12 (clone 79,018, R&D systems), rabbit anti-human VAP-1 (polyclonal, Sigma-Aldrich), rabbit anti-human CD103 (clone er-ACT8, Abcam) or mouse anti-human E-cadherin (clone HECD-1, Abcam). BrightVision Poly-HRP from Immunologic (Duiven, the Netherlands), was used as secondary antibody, and incubated for 1 ​h at room temperature. Staining was visualized with ImmPACT DAB peroxidase substrate (Vector Laboratories, USA) for 8 ​min, followed by counterstain with hematoxylin. Ileum, tonsil, liver and colon tissue were used as positive controls for MAdCAM-1 and CD103, CXCL12, VAP-1 and E-cadherin respectively. As a negative control, slides were incubated with PBT instead of primary antibody.

Since MAdCAM-1 staining was very weak in FFPE liver sections compared to frozen sections, fresh frozen tissue was used when available [27]. Sections of 4,5 ​μm were fixated in ice cold acetone for 10 ​min and washed with PBS. Endogenous peroxidase was blocked with Bloxall solution (Vectorlabs, Burlingame, CA) for 10 ​min, after which sections were incubated for 10 ​min with PBT to block nonspecific binding sites. Diluted primary antibody (mouse anti-human MAdCAM-1 (clone 355G8, Invitrogen) was added and incubated overnight at 4 ​°C. BrightVision Poly-HRP from Immunologic (Duiven, the Netherlands), was used as secondary antibody, and incubated for 1 ​h at room temperature. Staining was visualized with ImmPACT DAB peroxidase substrate (Vector Laboratories, USA) for 8 ​min, followed by counterstain with haematoxillin. Again, ileum tissue was used as positive control for MAdCAM-1 expression, and slides were incubated with PBT instead of the primary antibody to act as a negative control.

### 2.3 Evaluation of histological staining

All stainings were scored in a blinded manner by an expert hepatopathologist (JV) using a semiquantitative scale. Staining was defined as present or not present in a specific cell type depending on the marker, e.g. for liver biliary epithelium, portal endothelium or sinusoidal endothelium and for colon epithelial or endothelial cell staining. If possible, the intensity of each staining was graded as 0 (no staining), 1 (weak staining), 2 (moderate staining) or 3 (strong staining). For MAdCAM-1 a H-score was calculated with the following formula: *[3 x percentage of strongly staining cells]* ​+ ​*[2 x percentage of moderately staining cells]* ​+ ​*[percentage of weakly staining cells]*. This formed a score ranging from 0 to 300 [28].

### 2.4 In situ hybridization

For in situ hybridization, a RNAscope® assay was used according to the manufacturer’s protocol [29]. In short, FFPE slides were baked in a dry oven for 1 ​h at 60 ​°C, after which slides were deparaffinized in fresh xylene and dehydrated in an ethanol series. Tissue sections were then incubated with H_2_O_2_ for 10 ​min, followed by target retrieval at 98–102 ​°C for 30 ​min. Tissue sections were treated with Protease Plus (Advanced Cell Diagnostics, Hayward, CA) and incubated at 40 ​°C for 30 ​min in a HybEZ hybridization oven (Advanced Cell Diagnostics, Hayward, CA). The signal was detected using a Fast RED solution, causing a red immunofluorescent signal. Slides were then mounted with SlowFade Gold Antifade mountant containing 4′,6-diamidino-2-phenylindole (DAPI) (Life Technologies) to stain the nuclei. In order to perform double staining, slides were additionally incubated for 1 ​h with a primary antibody followed by 30 ​min incubation with a secondary antibody labeled with AF488 and then mounted with SlowFade Gold Antifade mountant containing DAPI (Life Technologies). HsPPIB and hsDapB probes were used as a positive and negative control, respectively. Other probes used were HsCCL25, HsCCL28 and HsITGB7.

### 2.5 Evaluation of in situ hybridization

Representative pictures of inflammatory sites were taken of each liver sample (3 in case of biopsies, 5 in case of resection specimens). All CD3^+^ *ITGB7*^+^ and CD3^+^ *ITGB7*^-^ cells were manually counted using ImageJ software, after which the fraction of ITGB7 positive cells to total CD3 positive cells was calculated. Expression of CCL28 on biliary epithelial cells was scored using a scale ranging from 0 to 3 on 3 to 5 representative fields.

### 2.6 Quantitative real-time polymerase chain reaction

RNA was extracted from liver tissue using the Bioline ISOLATE II RNA mini kit (GC biotech B.V. Alphen a/d Rijn, the Netherlands) according to the manufacturer’s instructions. RNA concentration was measured using the Nanodrop 1000 spectrophotometer (Nanodrop Technologies, Wilmington, DE, USA). cDNA was synthesized using the Revertaid first strand cDNA synthesis kit (Fermentas, St. Leon-Rot, Germany). A quantitative polymerase chain reaction (PCR) was performed using SensiFAST SYBR No-ROX (GC Biotech B·V.) on a BioRad (CFX96 real-time qPCR thermocycler) to analyze expression levels of *MAdCAM-1*, *ITGB7*, *CCL25*, *CCL28*, *VAP-1,* and *CK7*. For normalization, human reference genes β2 Macroglobuline (*B2M*) and *β-Actin (ACTB)* were selected after analysis for stability in geNorm [30]. Primers (synthesized by Sigma or obtained from Qiagen) are listed in Supplementary Table 3.

### 2.7 Qiagen PCR array

Gene expression profile of 96 genes encoding for inflammatory cytokines and receptors was assessed by a human RT^2^ Profiler PCR Arrays purchased from Qiagen and performed according to manufacturer’s protocol. RNA was obtained from colon biopsies of patients with PSC-IBD (n ​= ​18) and patients with IBD (n ​= ​11). For normalization, human reference genes hypoxanthine phosphoribosyltransferase 1 *(HPRT1)* and *ACTB* were used.

### 2.8 Statistical analysis

Patient characteristics are expressed as median and interquartile range (25th-75th percentile). Dichotomous variables are expressed as percentage (%) of the cohort. Differences between groups were calculated with Chi-square test or Fisher’s Exact test for categorical variables and Mann-Whitney *U* test for numerical variables. Differences in presence of staining between groups were calculated with Mann-Whitney U or Kruskal Wallis for two or more groups respectively, with Dunn’s correction for multiple testing. Statistical analyses were performed using SPSS version 24 software (SPSS, Chicago, IL) or GraphPad Prism 8. A p-value <0.05 was considered statistically significant.

## 3 Results

### 3.1 MAdCAM-1 is aberrantly expressed in PSC-IBD liver compared to control liver

**Fig. 1:**
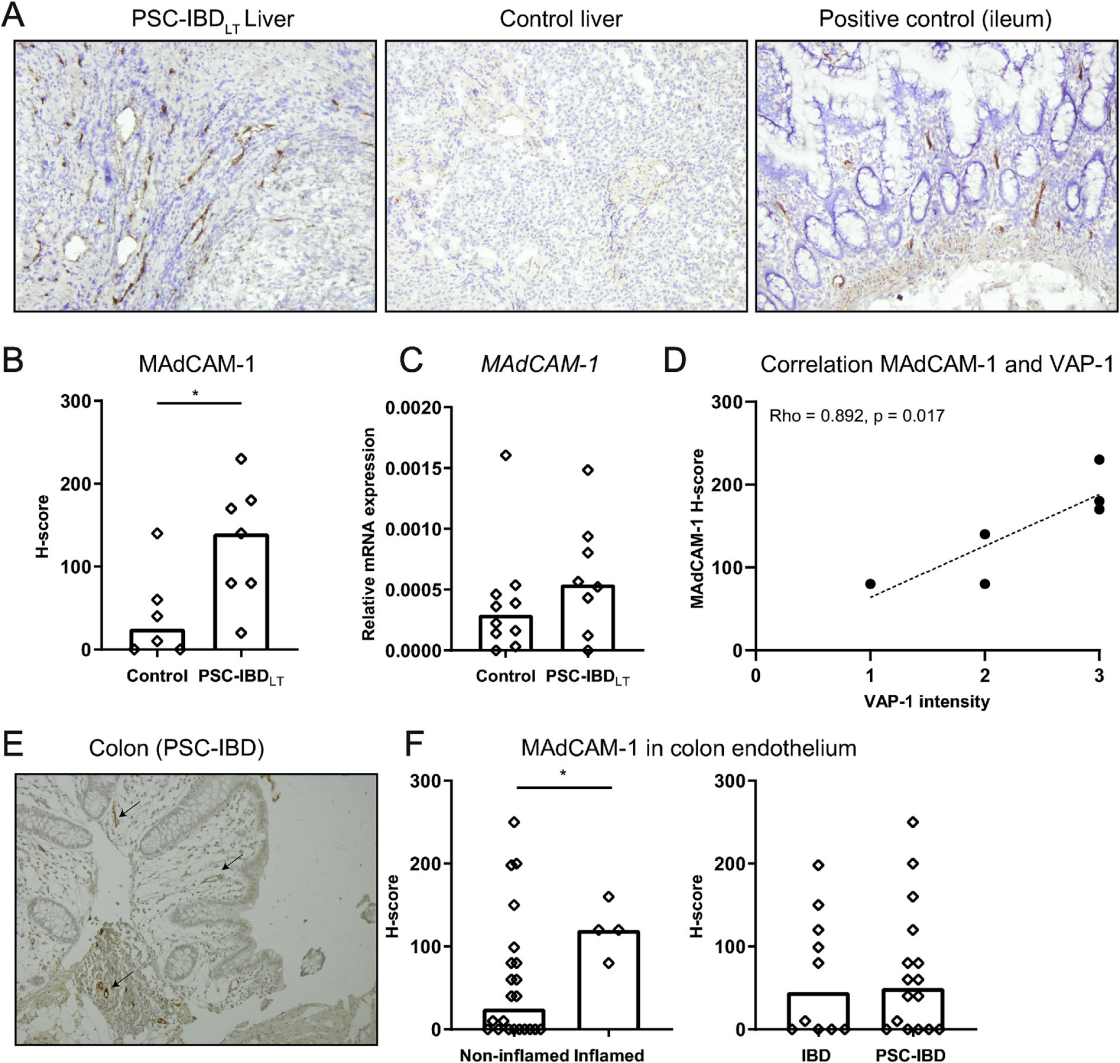
**MAdCAM-1 is present in livers of PSC-IBD patients and not increased in colonic tissue of PSC-IBD patients compared to IBD patients**. A: Immunohistochemistry of MAdCAM-1 on frozen sections, 10x magnification. Representative pictures of long-term PSC-IBD (PSC-IBD_LT_) liver (left) and control liver (middle). Ileum tissue was used as positive control (right). B: H-score of immunohistochemical MAdCAM-1 staining in control liver (n ​= ​6) and PSC-IBD_LT_ ​liver (n ​= ​7). H-score was calculated as [*3 x percentage of strongly staining cells*] ​+ ​[*2 x percentage of moderately staining cells*] ​+ ​[*percentage of weakly staining cells*]. C: Relative mRNA expression of ​*MAdCAM-1* ​in livers of control patients (n ​= ​10) and PSC-IBD_LT_ ​patients (n ​= ​8). D: Spearman’s Rho correlation between MAdCAM-1 H-score and VAP-1 intensity in livers of patients with PSC-IBD_LT_ ​(n ​= ​6). E: Representative picture of immunohistochemistry of MAdCAM-1 on FFPE colon sections, 10x magnification. Expression of MAdCAM-1 on endothelium of a patient with PSC-IBD (arrows). F: Difference in MAdCAM-1 staining between endoscopically inflamed colonic biopsies (n ​= ​4) and non-inflamed biopsies (n ​= ​22) of PSC-IBD and IBD patients (left panel). H-score of immunohistochemical MAdCAM-1 staining on endothelium in colon of IBD patients (n ​= ​10) and PSC-IBD patients (n ​= ​16) (right panel).Data presented as median. Statistical testing was performed using the Mann-Whitney ​*U* ​test for comparisons between groups. A p-value <0.05 was considered statistically significant (∗p ​< ​0.05).

Previous reports have shown aberrant expression of MAdCAM-1 in livers of end-stage PSC patients [6,[13], [14], [15]]. Optimization of MAdCAM-1 tissue staining on paired FFPE and frozen liver sections of 7 PSC patients showed that MAdCAM-1 staining yielded a large discrepancy (1/7 positive slides in FFPE and 7/7 positive slides in frozen tissue). Therefore, further analysis was carried out on frozen sections. Immunohistochemistry on frozen livers of PSC-IBD_LT_ showed that MAdCAM-1 was expressed on portal endothelial cells in the liver, but not on sinusoidal endothelium (Fig. 1A). The proportion of endothelial cells staining positive for MAdCAM-1 as calculated by the H-score was significantly higher in liver tissue of patients with PSC-IBD compared to control liver tissue (p ​= ​0.022; Fig. 1A and B). There was no difference in MAdCAM-1 staining in the liver between patients with concomitant UC or CD (data not shown). Relative mRNA expression in whole liver tissue did not show a significant difference (p ​= ​0.245; Fig. 1C).

Expression of MAdCAM-1 can be regulated via oxidase activity of adhesion molecule VAP-1 [16]. VAP-1 expression on portal endothelium tended to be lower in PSC-IBD_ST_ compared to control liver (median intensity 1.5 vs 3 respectively, p ​= ​0.072), but did not differ between PSC-IBD_LT_ and control liver (median intensity 2.5 vs 2.0 respectively, p ​= ​0.429, Supplementary Fig. 1A and B). However, there was a positive correlation between VAP-1 staining and MAdCAM-1 staining in PSC-IBD_LT_ liver (Spearman’s Rho 0.892, p ​= ​0.017; Fig. 1D).

During active inflammation, expression of MAdCAM-1 is increased in ileum and colon of UC and CD patients compared to controls [6,31]. Since expression of MAdCAM-1 was present in the livers of PSC patients, we hypothesized that there was also an increase in MAdCAM-1 expression in the intestine of patients with PSC-IBD, leading to a more general increased homing potential of α4β7^+^ T-cells in patients with PSC-IBD. Immunohistochemical staining showed presence of MAdCAM-1 on high endothelial vessels (Fig. 1E). Although expression was significantly higher in inflamed tissue compared to non-inflamed tissue in general (p ​= ​0.046; Fig. 1F), we found no difference in MAdCAM-1 expression in colonic tissue of the ascending colon of patients with PSC-IBD compared to patients with IBD (p ​= ​0.927; Fig. 1F). Additional transcriptional analysis in these biopsies did not show a clear distinction between these two groups in a panel of 96 genes (Supplementary Fig. 2). Only 10 out of 96 genes tested differed more than 2 fold in the PSC-IBD group compared to the IBD group. The majority was upregulated in the PSC-IBD group (9/10) however, none of them showed a statistical relevant difference. Notably, endoscopic inflammation was only present in 2 out of 18 samples analyzed, which showed upregulation of several genes (Supplementary Fig. 2).

### 3.2 The proportion of beta7 positive T-cells is increased in livers of patients with long-term PSC

**Fig. 2:**
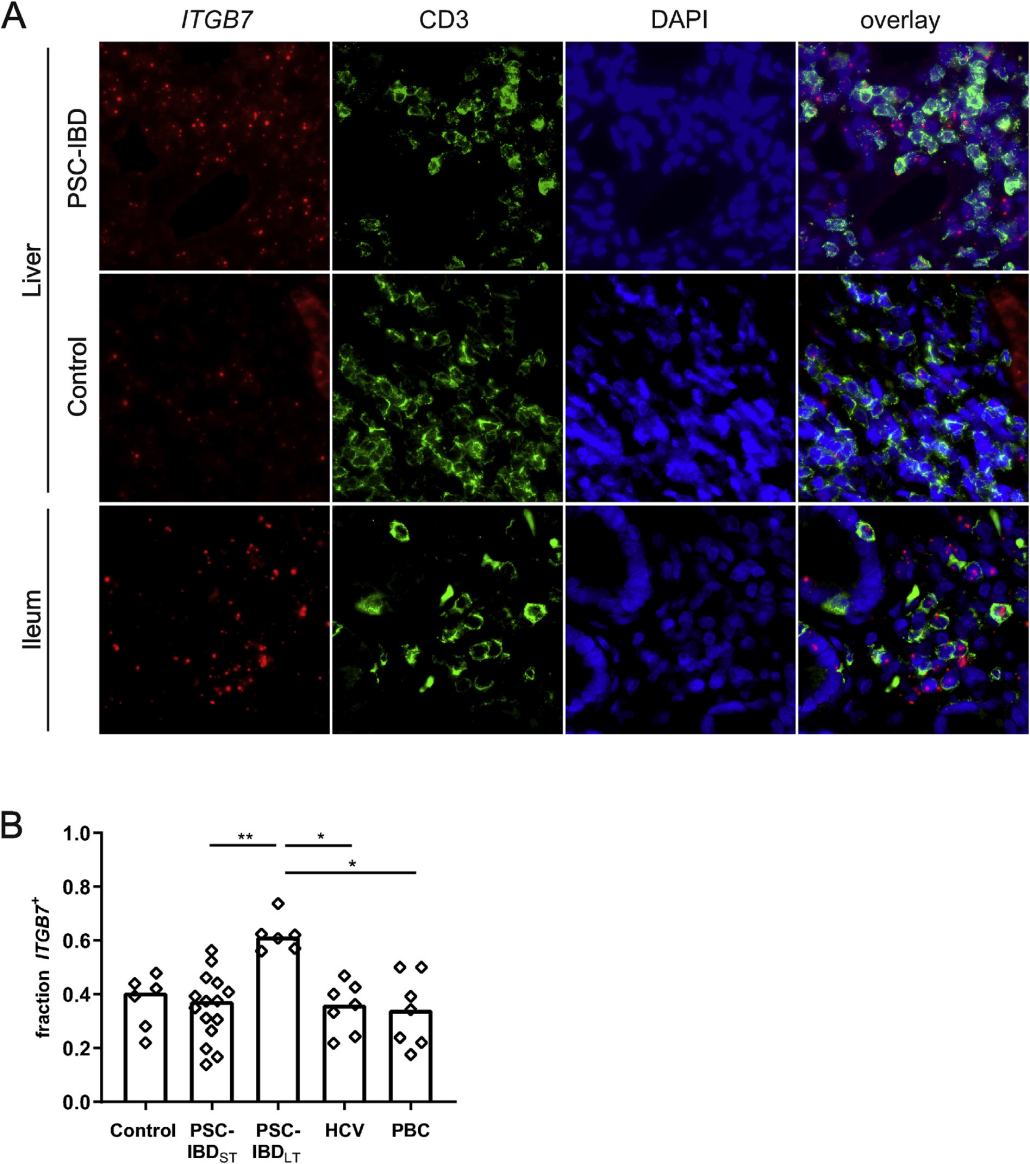
**The proportion of β7 positive T-cells is higher in livers of long-term PSC-IBD patients compared to short-term PSC-IBD livers and other liver diseases**. A: In situ hybridization (ISH) and immunofluorescent (IF) staining of ​*ITGB7* ​and CD3 respectively on FFPE sections, 40x magnification. Representative pictures of PSC-IBD liver (upper panel) and control liver (middle). Ileum tissue was used as a positive control (lower panel). B: Fraction of CD3^+^ ​T-cells that show mRNA expression of ​*ITGB7* ​in control liver (n ​= ​6), PSC-IBD_ST_ ​liver (n ​= ​15), PSC-IBD_LT_ ​liver (n ​= ​6), HCV liver (n ​= ​7) and PBC liver (n ​= ​7) using combined ISH and IF. Statistical testing was performed using Kruskal Wallis with Dunn’s correction for multiple testing. Data presented as median. A p-value <0.05 was considered statistically significant (∗p ​< ​0.05, ∗∗P ​< ​0.01).

The increased expression of MAdCAM-1 in PSC livers may lead to an increased influx of α4β7 positive T-cells. To assess this, in situ hybridization of *ITGB7* (the gene encoding for β7) was combined with immunofluorescent staining of CD3 in order to look for expression of *ITGB7* mRNA specifically in T-cells. Ileal tissue was used as a positive control, and showed clear expression of *ITGB7* mRNA in T-cell infiltrates as well as in intraepithelial T cells (Fig. 2A, lower panel). In the liver, there was no difference between PSC-IBD at time of diagnosis and controls (Fig. 2A and B). However, the proportion of CD3^+^ T-cells expressing *ITGB7* was significantly higher in PSC-IBD_LT_ livers compared to PSC-IBD_ST_ (p ​= ​0.004; Fig. 2A and B). To assess if this infiltration of β7 positive T-cells was disease-specific, we analyzed the expression of *ITGB7* in two other liver diseases. Liver tissue was obtained from seven patients with HCV as well as seven patients with PBC, both diseases characterized by dense, mononuclear cell infiltrates. The proportion of *ITGB7* positive lymphocytes in these conditions did not differ significantly from that seen in controls (Fig. 2B). The difference in *ITGB7* expression between short-term and long-term PSC suggested a role for disease duration or stage. Overall, there was a positive correlation between *ITGB7* expression and PSC disease duration (Spearman’s Rho 0.700, p ​< ​0.001; Supplementary Fig. 3A). Fibrosis stage was significantly different between patients with short-term and long-term PSC-IBD, with a median Ludwig stage of 2 in the livers of PSC-IBD_ST_ (range 0–4) and 4 in the livers of PSC-IBD_LT_ (range 3–4; p ​= ​0.010) (Table 1). When all short term and long term liver samples were lumped together, there was a positive correlation between Ludwig stage and *ITGB7* expression (Spearman’s Rho 0.545, p ​= ​0.011; Supplementary Fig. 3B). In multivariate linear regression analysis, disease duration but not Ludwig stage was a predictor for *ITGB7* expression (p ​= ​0.011 and p ​= ​0.802, respectively).

### 3.3 CD103^+^ cells are present in a subset of short-term PSC livers

**Fig. 3:**
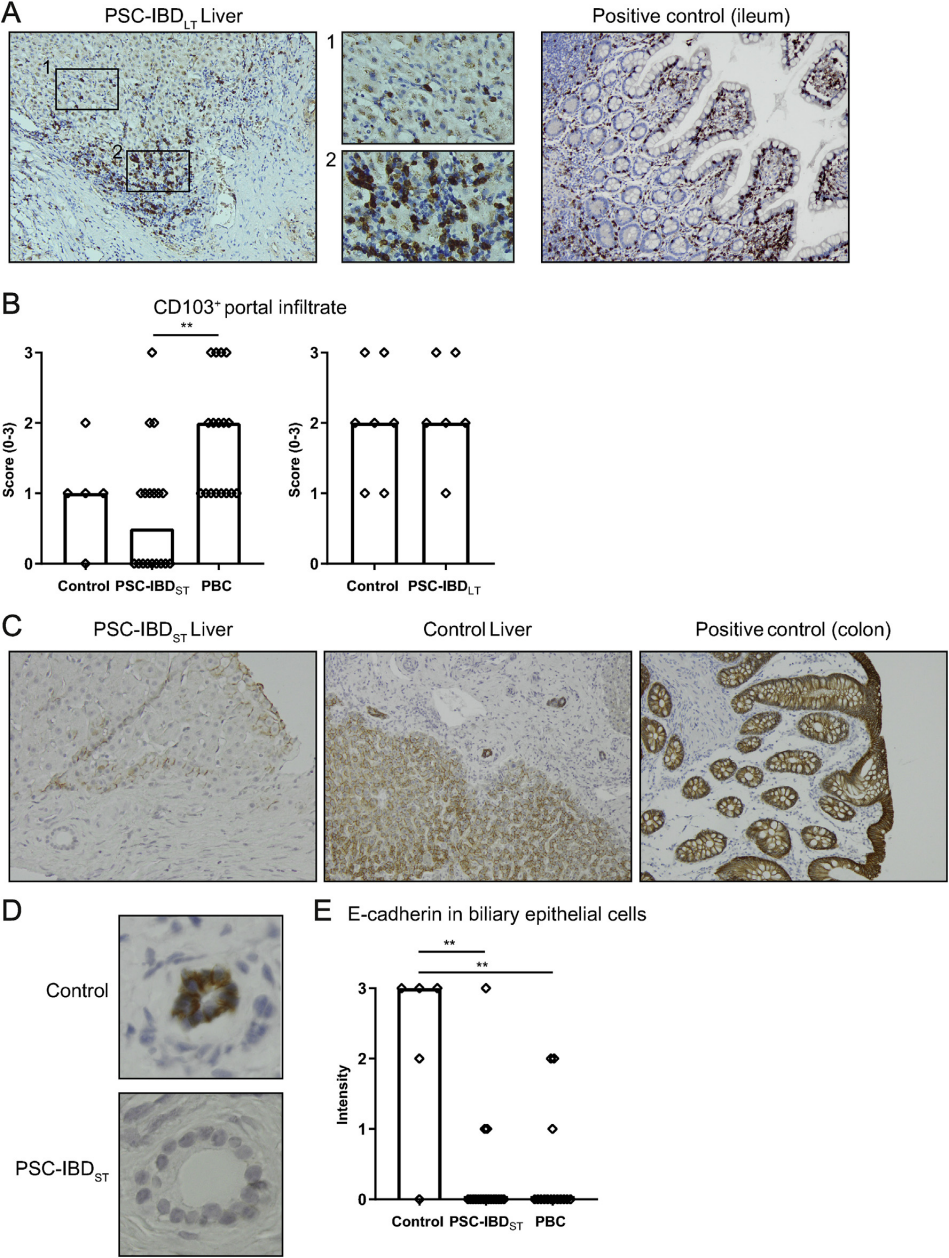
**CD103**^**+**^**cells are present in a subset of short-term PSC livers.** A: Immunohistochemistry of CD103 on FFPE sections, 10x magnification. Ileum tissue was used as positive control (left), representative picture of a long-term PSC-IBD (PSC-IBD_LT_) liver. 40x magnification of positive cells in the sinusoidal area (1) and in portal area (2). B: Staining (0–3) of CD103^+^ cells in portal infiltrates of controls (n ​= ​5), PSC-IBD_ST_ (n ​= ​18) and PBC (n ​= ​17) (left) and controls (n ​= ​7) and PSC-IBD_LT_ livers (n ​= ​6) (right). C: Immunohistochemistry of E-cadherin on FFPE sections, 10x magnification. Representative picture of a PSC-IBD liver (left) and control liver (middle). Colon tissue was used as positive control (right). D: Biliary epithelial cells expressing E-cadherin in control tissue, loss of E-cadherin in short-term PSC-IBD liver, 40x magnification. E: Staining intensity (0–3) of E-cadherin in biliary epithelial cells of control patients (n ​= ​5), PSC-IBD patients with short-term disease (n ​= ​18) and PBC patients (n ​= ​15). Statistical testing was performed using the Mann-Whitney *U* test for comparing 2 groups and Kruskal Wallis for comparing more than 2 groups. Data presented as median. A p-value <0.05 was considered statistically significant (∗p ​< ​0.05, ∗∗P ​< ​0.01).

Integrin β7 can form two heterodimers, α4β7 and αEβ7. It has been proposed that memory T-cells expressing α4β7 upregulate integrin αE (CD103) upon recruitment to the liver to remain tissue resident, similar to the expression observed in the ileum [32]. Indeed, in ileum tissue used as a positive control, we could confirm expression of CD103 by intra-epithelial cells presumed to be lymphocytes (Fig. 3A, right panel). In liver tissue, cells expressing CD103 were present both in portal as well as in sinusoidal areas (Figs. 3A–1, 3A-2). Distribution was very heterogeneous with varying numbers of positive cells in different portal fields within the same patient. CD103^+^ cells were more abundant in PBC livers compared to PSC-IBD livers; CD103 was not expressed in the portal infiltrates of half of the short-term PSC biopsies, whereas CD103 was present in all PBC biopsies (Fig. 3B). In the livers of patients with PSC-IBD_LT_, CD103^+^ cells were present in all livers, in which intensity was comparable to control livers (Fig. 3B). Of note, in some livers also Kupffer cells expressed CD103.

The receptor of αE, E-cadherin, is constitutively expressed by intestinal epithelial cells (Fig. 3C, right panel), and did not differ between colons of PSC-IBD patients and IBD patients (data not shown). In normal liver tissue, hepatocytes as well as biliary epithelial cells express E-cadherin (Fig. 3C, middle panel). In patients with short-term PSC-IBD, expression of E-cadherin on biliary epithelial cells was less present compared to control liver, which was also the case in PBC livers (Fig. 3D and E). Yet in long-term disease, this was difficult to compare, as there was prominent bile duct loss in the majority of livers (bile duct loss in more than two-third of portal tracts in 4/6 livers). E-cadherin was however expressed by reactive ductules. There was a modest positive correlation between loss of E-cadherin expression and loss of CD103^+^ cells in the portal area (Spearman’s Rho 0.336, p ​= ​0.017).

### 3.4 CCL28 expression by biliary epithelial cells is increased in short-term PSC

**Fig. 4:**
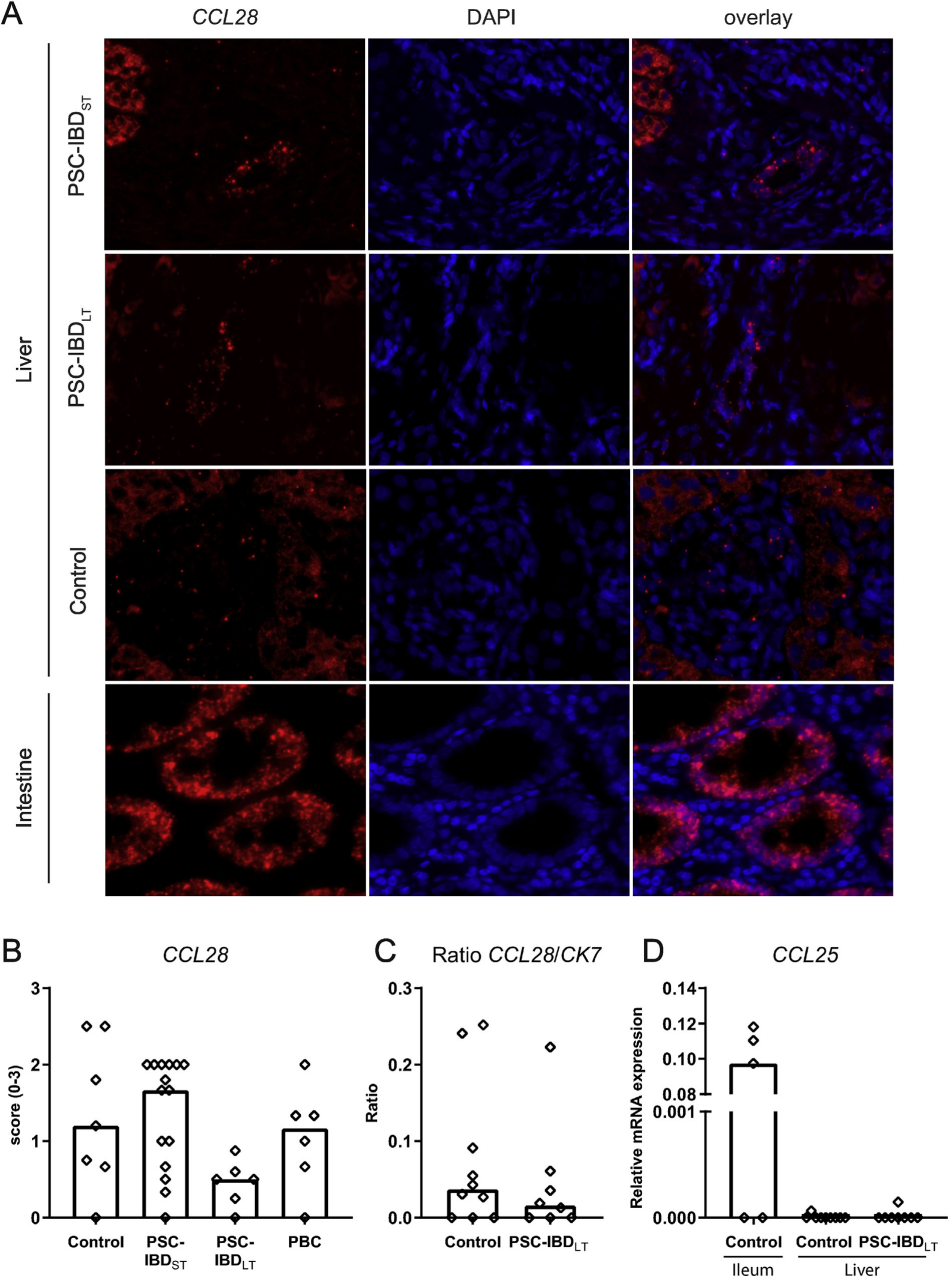
***CCL2*****8 mRNA expression differs between long-term and short-term PSC livers.** A: In situ hybridization (ISH) of *CCL28* on FFPE sections, 40x magnification. Representative pictures of short-term PSC-IBD (PSC-IBD_ST_), long-term PSC-IBD (PSC-IBD_LT_) and control liver. Colon tissue was used as a positive control (lower panel). B: Presence of *CCL28* (score 0–3) in biliary epithelium of livers from controls (n ​= ​7), PSC-IBD_ST_ (n ​= ​15), PSC-IBD_LT_ (n ​= ​6) and PBC (n ​= ​6) patients. C: Ratio of *CCL28*/*CK7* mRNA expression in livers of control patients (n ​= ​10) and PSC-IBD_LT_ patients (n ​= ​8). D: mRNA expression of *CCL25* in ileum tissue (n ​= ​5) and livers of control patients (n ​= ​9) and long-term PSC-IBD patients (n ​= ​8). Statistical testing was performed using the Mann-Whitney *U* test for comparing 2 groups, Kruskal-Wallis with Dunn’s correction for multiple testing for comparing more than 2 groups. Data presented as median. A p-value <0.05 was considered statistically significant.

There are different chemokines involved in trafficking of T-cells towards the bile ducts. As described earlier, *CCL28* was expressed by biliary epithelial cells in the liver (Fig. 4A) [19]. In early stage PSC, expression of *CCL28* tended to be higher than that seen in controls and PBC patients, although this failed to reach statistical significance (Fig. 4B). In long-term PSC-liver, expression of *CCL28* was decreased. We investigated whether this was potentially due to the loss of bile ducts in long-term PSC. As noted previously, bile duct loss was present in the majority of long-term PSC-IBD livers (4/6 livers). On the contrary in the short-term PSC-IBD livers, the majority contained no bile duct loss (7/15) or bile duct loss in less than one-third of portal tracts (8/15). Nevertheless, there was no difference in *CCL28* expression between livers with and without bile duct loss in PSC-IBD_LT_, indicating that reactive bile ducts express CCL28, but in a lower amount than in PSC-IBD_ST_. Additionally, mRNA expression of *CK7*, a cholangiocyte marker, was assessed in liver tissue, showing no difference in the ratio between relative mRNA expression of *CCL28* and *CK7* in PSC-IBD_LT_ livers compared to control livers (p ​= ​0.410; Fig. 4C).

CCL25, which is highly present in ileal epithelial cells, was only very scantly present in liver tissue of both PSC liver and control livers (pictures not shown). This was confirmed in relative mRNA expression of whole liver tissue compared to ileum tissue (Fig. 4D).

### 3.5 Biliary epithelium as well as portal endothelium expresses CXCL12 in livers of PSC patients and controls

**Fig. 5:**
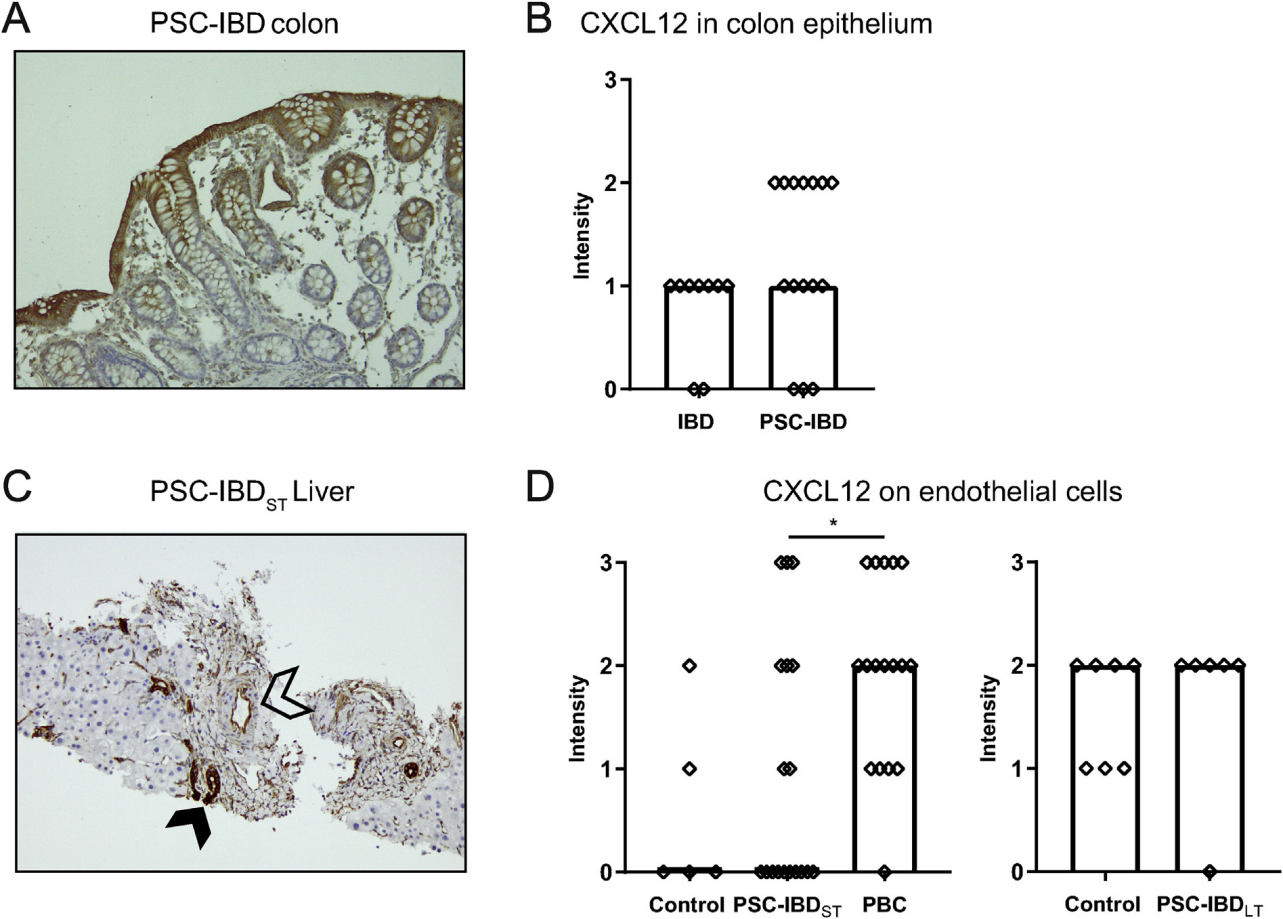
**CXCL12 is highly expressed by biliary epithelial cells, as well as on endothelial cells in PSC liver.** A: Representative picture of immunohistochemistry of CXCL12 on FFPE colon sections, 10x magnification, PSC-IBD colon. B: Staining intensity (0–3) of CXCL12 on colon epithelium of IBD patients (n ​= ​9) and PSC-IBD patients (n ​= ​15). C: Representative picture of immunohistochemistry of CXCL12 on FFPE liver sections, 10x magnification, short-term PSC-IBD (PSC-IBD_ST_) liver. Positive biliary epithelial cells are marked with the filled arrow, positive endothelial cells with the open arrow. D: Staining intensity (0–3) of CXCL12 on liver tissue of controls (n ​= ​5), PSC-IBD_ST_ livers (n ​= ​18) and PBC livers (n ​= ​17) (left) and control livers (n ​= ​7) and long-term PSC-IBD (PSC-IBD_LT_) livers (n ​= ​6) (right). Statistical testing was performed using the Mann-Whitney *U* test for comparing 2 groups and Kruskal Wallis with Dunn’s correction for multiple testing for comparing more than 2 groups. Data presented as median. A p-value <0.05 was considered statistically significant (∗p ​< ​0.05).

Epithelial expression of CXCL12 is increased in the intestine of patients with IBD compared to controls, which is even more pronounced in active inflammation [33]. CXCL12 expression was assessed in colonic tissue as well as in liver tissue of PSC-IBD patients using immunohistochemistry. Intestinal epithelium expressed CXCL12, showing a gradient from basal to apical (Fig. 5A). Staining intensity tended to be higher in PSC-IBD colon compared to colon of IBD patients, despite the absence of endoscopic inflammation in the majority of biopsies in both groups (p ​= ​0.083; Fig. 5B). In liver tissue, biliary epithelial cells (BECs) from pre-existent bile ducts as well as reactive ductules expressed CXCL12 in both PSC and PBC as well as in controls (Fig. 5C, filled arrow). Intensity of CXCL12 staining on BECs did not differ between short-term PSC-IBD patients, PBC patients and controls as well as between long-term PSC-IBD patients and controls (data not shown). Additionally, in a subset of patients, endothelial cells also expressed CXCL12, predominantly portal (Fig. 5C, open arrow). There was no difference in CXCL12 expression by endothelial cells between PSC liver and control liver (both long-term and short-term PSC) (Fig. 5D). However, there was a significantly higher amount of livers with endothelial expression of CXCL12 in PBC livers compared to PSC-IBD_ST_ livers (16/17 (94.1%) and 8/18 (44%) respectively, p ​= ​0.003).

## 4 Discussion

The presence of adhesion molecule MAdCAM-1 in livers of PSC patients is one of the leading findings supporting the hypothesis that gut-primed T-cells can infiltrate the liver in PSC. Whereas previous studies have shown that MAdCAM-1 is more frequently found in PSC patients compared to controls [[13], [14], [15]], we now showed that the extent of tissue expressing MAdCAM-1 is much larger in PSC liver compared to control liver. We could not confirm this finding with relative mRNA expression due to the fact that it is impossible to specifically correct for the amount of portal vascular endothelium in particular in the liver specimens as we did in the immunostainings. Although MAdCAM-1 can be upregulated by VAP-1, we did not see an increase in VAP-1 expression in the PSC livers compared to control livers as described by Trivedi et al. [17]. This could be explained by the presence of secondary sclerosing cholangitis in the control livers, showing signs of fibrosis (Ludwig stage 2–4) [34]. We did however find a positive correlation between VAP-1 and MAdCAM-1.

Integrin α4β7 is present on up to 90% of lymphocytes in the small bowel, and on around 25% of lymphocytes in the colon [9]. Ponsioen et al. [12] have shown that the infiltrates in non-endstage PSC liver contain significantly more α4β7^+^ lymphocytes than control liver, which was confirmed by Grant et al. [13] in end-stage PSC liver. We support these findings by showing that a higher percentage of CD3^+^ T-cells expressing *ITGB7* was present in long-term PSC-IBD. On the contrary, expression levels of *ITGB7* in short-term PSC-IBD were comparable to controls. As β7 is part of two heterodimers, integrin α4β7 and integrin αEβ7, we were not able to distinguish between these two types of T-cells in this analysis. We found no significant difference in αEβ7^+^ T-cells between long-term PSC-IBD liver and control liver. Hence, we suggest that the difference found in ITGB7^+^ cells in long-term PSC-IBD is due to the presence of α4β7^+^ T-cells. This could indicate that infiltration of α4β7^+^ T-cells is increased predominantly in end-stage disease. This is also in line with findings by Ala et al. showing that hepatic *MAdCAM-*1 mRNA expression is upregulated in cirrhosis, so long-term PSC [15]. Unfortunately, a specific staining for α4β7 is not available for FFPE procured tissue.

Interestingly, the receptor of αE, E-cadherin, was less present on biliary epithelial cells in livers of PSC-IBD patients, as well as on BECs of PBC patients. This is in line with previous findings showing loss of E-cadherin expression in the epithelial cells of medium-sized bile ducts of PSC patients [21]. E-cadherin is expressed at epithelial adherent junctions and has different functions in maintaining homeostasis. In long-term PSC-IBD, E-cadherin was predominantly expressed by reactive ductules, as there was progressive bile duct loss present in the majority of PSC-IBD livers.

Chemokine CCL25 aids in the *trans*-endothelial migration of gut-homing T-cells, so enhanced expression will promote infiltration of T-cells. In our study, we could not confirm the aberrant expression of CCL25 in end-stage PSC liver as described by Eksteen et al. [8]. Previous research has shown that in addition to CCL25, also CCL28 is able to promote the adhesion of α4β7^+^ lymphocytes to MAdCAM-1 [7]. CCL28, which is highly expressed by colonic epithelium [11], could be of particular interest in the gut-liver axis in PSC, as the majority of PSC patients has colonic instead of ileal disease. Its receptor CCR10 is expressed on B- and T-cells in the small and large intestine. In inflamed human liver (including PSC and PBC), a subset of regulatory T-cells (Tregs) expresses CCR10, possibly contributing to limitation of inflammation [19]. We confirmed expression of CCL28 in PSC livers, which was -interestingly- most prominent in short-term PSC liver and less abundant in long-term PSC liver. Possibly, Tregs are recruited to the liver to dampen inflammation and limit the tissue damage, but this response cannot be maintained if the damage progresses. Amplification of CCL28 expression has been associated with both proliferation of reactive bile ducts (ductular reaction) in liver, as well as with epithelial inflammation in gut epithelium [19,35]. We did not specifically look at presence of reactive bile ducts, but in our PSC-IBD livers, we did not see a clear difference in CCL28 expression between livers with and without bile duct loss or with different degrees of bile duct loss.

Expression of the chemokine CXCL12 on biliary epithelial cells is low in normal liver, but enhanced in inflammatory diseases of the liver such as PSC, PBC and HCV [8,18]. We did not find a difference in biliary epithelial expression of CXCL12 between control liver and short- or long-term PSC-IBD, but as a proportion of our control liver tissue was derived from patients operated on for distal cholangiocarcinoma with subsequent secondary cholangitis and fibrosis, this could have led to a higher expression in the controls. The similar expression of CXCL12 on BECs from both PSC-IBD and PBC livers supports this. Interestingly, in contrast to previous reports on PSC liver, we did observe expression of CXCL12 by endothelial cells. As CXCL12 is able to promote binding of α4β7^+^ lymphocytes to MAdCAM-1 *in vitro*, this could indicate that CXCL12 is involved in promoting entering of gut-primed lymphocytes to the liver, though this seems to be not specific for PSC [7,36]. Epithelial expression of CXCL12 in gut tissue is increased in IBD patients versus controls, in particular during inflammation [33]. When comparing IBD patients with and without PSC, staining intensity was significantly higher in the colonic tissues of patients with PSC. The gradient from apical to basal in epithelial cells could be an indication that CXCL12 is used to direct lymphocytes from the high endothelial venules into the lamina propria, yet we did not observe a similar pattern in liver tissue.

Transcriptome analysis of colonic tissue showed only limited differences between PSC-IBD and IBD colons in a panel of 96 genes associated with human inflammatory cytokines and receptors. A recent study by Quraishi and Acharjee et al. showed 1692 genes that were differentially expressed between colons of 10 PSC-IBD and 10 UC patients [37]. They found an upregulation of genes associated with bile acid signaling pathways, and a downregulation of processes associated with immunological responses. Interestingly, they showed no differences in immunological subsets between colons of patients with PSC-IBD and UC. Possibly, this could indicate why we only saw limited differences, at least in endoscopically non-inflamed colons.

One important limitation of this study was the absence of fresh frozen liver tissue of short-term PSC patients to specifically look at presence of MAdCAM-1 and α4β7 in early stage. Furthermore, the sample sizes were small, which could have led to a lack of power to show differences. The immunohistochemical stainings included were technically difficult. Internal negative control slides and validated positive controls, available for all cases, were used for quality assurance.

In summary, in this descriptive study we confirmed the aberrant expression of MAdCAM-1 in livers of patients with PSC-IBD. The expression of MAdCAM-1 by portal and not sinusoidal endothelium, as well as presence of other chemokines and integrins like CCL28 and β7 in and around the bile ducts suggest that the inflammatory trigger in PSC originates in the portal area, i.e. in the bile ducts, thereby provoking cholangitis and not hepatitis. We did not observe clear differences in colon tissue of PSC-IBD versus IBD patients, suggesting that the aberrant homing predominantly takes place in the liver, and not in both liver and intestine, at least when there is no active inflammation present in the intestine. As expected, expression of certain homing markers differed between short-term and long-term PSC-IBD, but changes in short-term PSC livers appeared less prominent than in long-term PSC. The upregulation of β7 seen in long-term PSC was not present in short-term PSC suggesting that homing of α4β7^+^ lymphocytes appears in a later phase, when there is already damage of the liver. The differences found between short-term and long-term PSC indicate that the immune responses differ between disease stages. Our findings support the gut-homing hypothesis linking gut and liver in PSC-IBD.

## Funding

This work was supported by an investigator-initiated grant from Takeda.

## CRediT authorship contribution statement

**Manon de Krijger:** Conceptualization, Formal analysis, Investigation, Writing - original draft. **Thijmen Visseren:** Investigation, Resources, Writing - review & editing. **Manon E. Wildenberg:** Conceptualization, Formal analysis, Writing - review & editing. **Gerrit K.J. Hooijer:** Resources. **Monique M.A. Verstegen:** Resources, Writing - review & editing. **Luc J.W. van der Laan:** Resources, Supervision. **Wouter J. de Jonge:** Conceptualization, Supervision. **Joanne Verheij:** Conceptualization, Investigation, Resources, Writing - review & editing, Supervision. **Cyriel Y. Ponsioen:** Conceptualization, Writing - review & editing, Supervision.

## Declaration of competing interest

C.Y. Ponsioen received research grants form Takeda, speaker’s fees from Takeda, Tillotts, and Abbvie, and consultancy fees from Takeda and Pliant. All other authors declare that they have no known competing financial interests or personal relationships that could have appeared to influence the work reported in this paper.

## References

[1] Boonstra K., Weersma R.K., van Erpecum K.J., Rauws E.A., Spanier B.W., Poen A.C. (2013). Population-based epidemiology, malignancy risk, and outcome of primary sclerosing cholangitis. Hepatology.

[2] Sorensen J.O., Nielsen O.H., Andersson M., Ainsworth M.A., Ytting H., Belard E. (2018). Inflammatory bowel disease with primary sclerosing cholangitis: a Danish population-based cohort study 1977-2011. Liver Int. : official journal of the International Association for the Study of the Liver.

[3] Jorgensen K.K., Grzyb K., Lundin K.E., Clausen O.P., Aamodt G., Schrumpf E. (2012). Inflammatory bowel disease in patients with primary sclerosing cholangitis: clinical characterization in liver transplanted and nontransplanted patients. Inflamm. Bowel Dis..

[4] Adams D.H., Eksteen B. (2006). Aberrant homing of mucosal T cells and extra-intestinal manifestations of inflammatory bowel disease. Nat. Rev. Immunol..

[5] Iwata M., Hirakiyama A., Eshima Y., Kagechika H., Kato C., Song S.Y. (2004). Retinoic acid imprints gut-homing specificity on T cells. Immunity.

[6] Briskin M., Winsor-Hines D., Shyjan A., Cochran N., Bloom S., Wilson J. (1997). Human mucosal addressin cell adhesion molecule-1 is preferentially expressed in intestinal tract and associated lymphoid tissue. Am. J. Pathol..

[7] Miles A., Liaskou E., Eksteen B., Lalor P.F., Adams D.H. (2008). CCL25 and CCL28 promote alpha 4 beta7-integrin-dependent adhesion of lymphocytes to MAdCAM-1 under shear flow. Am. J. Physiol. Gastrointest. Liver Physiol..

[8] Eksteen B., Grant A.J., Miles A., Curbishley S.M., Lalor P.F., Hubscher S.G. (2004). Hepatic endothelial CCL25 mediates the recruitment of CCR9+ gut-homing lymphocytes to the liver in primary sclerosing cholangitis. J. Exp. Med..

[9] Kunkel E.J., Campbell J.J., Haraldsen G., Pan J., Boisvert J., Roberts A.I. (2000). Lymphocyte CC chemokine receptor 9 and epithelial thymus-expressed chemokine (TECK) expression distinguish the small intestinal immune compartment: epithelial expression of tissue-specific chemokines as an organizing principle in regional immunity. J. Exp. Med..

[10] Trivedi P.J., Bruns T., Ward S., Mai M., Schmidt C., Hirschfield G.M. (2016). Intestinal CCL25 expression is increased in colitis and correlates with inflammatory activity. J. Autoimmun..

[11] Ogawa H., Iimura M., Eckmann L., Kagnoff M.F. (2004). Regulated production of the chemokine CCL28 in human colon epithelium. Am. J. Physiol. Gastrointest. Liver Physiol..

[12] Ponsioen C.Y., Kuiper H., Ten Kate F.J., van Milligen de Wit M., van Deventer S.J., Tytgat G.N. (1999). Immunohistochemical analysis of inflammation in primary sclerosing cholangitis. Eur. J. Gastroenterol. Hepatol..

[13] Grant A.J., Lalor P.F., Hubscher S.G., Briskin M., Adams D.H. (2001). MAdCAM-1 expressed in chronic inflammatory liver disease supports mucosal lymphocyte adhesion to hepatic endothelium (MAdCAM-1 in chronic inflammatory liver disease). Hepatology.

[14] Hillan K.J., Hagler K.E., MacSween R.N., Ryan A.M., Renz M.E., Chiu H.H. (1999). Expression of the mucosal vascular addressin, MAdCAM-1, in inflammatory liver disease. Liver.

[15] Ala A., Brown D., Khan K., Standish R., Odin J.A., Fiel M.I. (2013). Mucosal addressin cell adhesion molecule (MAdCAM-1) expression is upregulated in the cirrhotic liver and immunolocalises to the peribiliary plexus and lymphoid aggregates. Dig. Dis. Sci..

[16] Liaskou E., Karikoski M., Reynolds G.M., Lalor P.F., Weston C.J., Pullen N. (2011). Regulation of mucosal addressin cell adhesion molecule 1 expression in human and mice by vascular adhesion protein 1 amine oxidase activity. Hepatology.

[17] Trivedi P.J., Tickle J., Vesterhus M.N., Eddowes P.J., Bruns T., Vainio J. (2018). Vascular adhesion protein-1 is elevated in primary sclerosing cholangitis, is predictive of clinical outcome and facilitates recruitment of gut-tropic lymphocytes to liver in a substrate-dependent manner. Gut.

[18] Terada R., Yamamoto K., Hakoda T., Shimada N., Okano N., Baba N. (2003). Stromal cell-derived factor-1 from biliary epithelial cells recruits CXCR4-positive cells: implications for inflammatory liver diseases. Lab. Invest..

[19] Eksteen B., Miles A., Curbishley S.M., Tselepis C., Grant A.J., Walker L.S. (2006). Epithelial inflammation is associated with CCL28 production and the recruitment of regulatory T cells expressing CCR10. J. Immunol..

[20] Lim S.P., Leung E., Krissansen G.W. (1998). The beta7 integrin gene (Itgb-7) promoter is responsive to TGF-beta 1: defining control regions. Immunogenetics.

[21] Nakagawa H., Hikiba Y., Hirata Y., Font-Burgada J., Sakamoto K., Hayakawa Y. (2014). Loss of liver E-cadherin induces sclerosing cholangitis and promotes carcinogenesis. Proc. Natl. Acad. Sci. U. S. A..

[22] de Krijger M., Wildenberg M.E., de Jonge W.J., Ponsioen C.Y. (2019). Return to sender: lymphocyte trafficking mechanisms as contributors to primary sclerosing cholangitis. J. Hepatol..

[23] Lennard-Jones J.E. (1989). Classification of inflammatory bowel disease. Scand. J. Gastroenterol. Suppl..

[24] European Association for the Study of the L. (2009). EASL Clinical Practice Guidelines: management of cholestatic liver diseases. J. Hepatol..

[25] Ludwig J., Dickson E.R., McDonald G.S. (1978). Staging of chronic nonsuppurative destructive cholangitis (syndrome of primary biliary cirrhosis). Virchows Arch. A Pathol. Anat. Histol..

[26] Nakanuma Y., Zen Y., Harada K., Sasaki M., Nonomura A., Uehara T. (2010). Application of a new histological staging and grading system for primary biliary cirrhosis to liver biopsy specimens: interobserver agreement. Pathol. Int..

[27] Scalia C.R., Boi G., Bolognesi M.M., Riva L., Manzoni M., DeSmedt L. (2017). Antigen masking during fixation and embedding, dissected. J. Histochem. Cytochem..

[28] Ishibashi H., Suzuki T., Suzuki S., Moriya T., Kaneko C., Takizawa T. (2003). Sex steroid hormone receptors in human thymoma. J. Clin. Endocrinol. Metab..

[29] Wang F., Flanagan J., Su N., Wang L.C., Bui S., Nielson A. (2012). RNAscope: a novel in situ RNA analysis platform for formalin-fixed, paraffin-embedded tissues. J. Mol. Diagn..

[30] Vandesompele J., De Preter K., Pattyn F., Poppe B., Van Roy N., De Paepe A. (2002). Accurate normalization of real-time quantitative RT-PCR data by geometric averaging of multiple internal control genes. Genome Biol..

[31] Arihiro S., Ohtani H., Suzuki M., Murata M., Ejima C., Oki M. (2002). Differential expression of mucosal addressin cell adhesion molecule-1 (MAdCAM-1) in ulcerative colitis and Crohn’s disease. Pathol. Int..

[32] Gorfu G., Rivera-Nieves J., Ley K. (2009). Role of beta7 integrins in intestinal lymphocyte homing and retention. Curr. Mol. Med..

[33] Dotan I., Werner L., Vigodman S., Weiss S., Brazowski E., Maharshak N. (2010). CXCL12 is a constitutive and inflammatory chemokine in the intestinal immune system. Inflamm. Bowel Dis..

[34] Weston C.J., Shepherd E.L., Claridge L.C., Rantakari P., Curbishley S.M., Tomlinson J.W. (2015). Vascular adhesion protein-1 promotes liver inflammation and drives hepatic fibrosis. J. Clin. Invest..

[35] Govaere O., Cockell S., Van Haele M., Wouters J., Van Delm W., Van den Eynde K. (2019). High-throughput sequencing identifies aetiology-dependent differences in ductular reaction in human chronic liver disease. J. Pathol..

[36] Wright N., Hidalgo A., Rodriguez-Frade J.M., Soriano S.F., Mellado M., Parmo-Cabanas M. (2002). The chemokine stromal cell-derived factor-1 alpha modulates alpha 4 beta 7 integrin-mediated lymphocyte adhesion to mucosal addressin cell adhesion molecule-1 and fibronectin. J. Immunol..

[37] Quraishi M.N., Acharjee A., Beggs A.D., Horniblow R., Tselepis C., Gkoutus G. (2020). A pilot integrative analysis of colonic gene expression, gut microbiota and immune infiltration in primary sclerosing cholangitis-inflammatory bowel disease: association of disease with bile acid pathways. J Crohns Colitis.

