# Supplementary material for: Characterization of gut-homing molecules in non-endstage livers of patients with primary sclerosing cholangitis and inflammatory bowel disease
Source: J Transl Autoimmun. 2020 Apr 9;3:100054. doi: 10.1016/j.jtauto.2020.100054 (PMC7388383; doi:10.1016/j.jtauto.2020.100054)
Supplement: Multimedia component 1 [file mmc1.pdf]

Supplementary material

Characterization of gut-homing molecules in non-endstage livers of patients with Primary Sclerosing Cholangitis and Inflammatory Bowel Disease

Manon de Krijger[a,b], Thijmen Visseren[c,e], Manon E. Wildenberg[a,b], Gerrit K.J. Hooijer[d], Monique M.A. Verstegen[e], Luc J.W. van der Laan[e], Wouter J. de Jonge[a], Joanne Verheij[d], Cyriel Y. Ponsioen[b*]

[a] Tytgat Institute for Liver and Intestinal Research, Amsterdam UMC, University of Amsterdam, Meibergdreef 69, 1105 BK, Amsterdam, The Netherlands

[b] Department of Gastroenterology and Hepatology, Amsterdam UMC, University of Amsterdam, Meibergdreef 9, 1105 AZ Amsterdam, The Netherlands

[c] Department of Gastroenterology and Hepatology, Erasmus MC-University Medical Center Rotterdam, Doctor Molewaterplein 40, 3015 GD, Rotterdam, The Netherlands

[d] Department of Pathology, Amsterdam UMC, University of Amsterdam, Meibergdreef 9, 1105 AZ, Amsterdam, The Netherlands

[e] Department of Surgery, Erasmus MC-University Medical Center Rotterdam, Doctor Molewaterplein 40, 3015 GD, Rotterdam, The Netherlands

Supplementary table 1 – Overview of used liver tissue and colonic tissue

1a. Used liver tissue

Group	Type	IBD type	Condition	Technique	Target
PSC-IBD	PSC-IBD_{ST}	20/20 UC	FFPE	IHC	VAP-1, CD103, E-cadherin, CXCL12
				ISH	*ITGB7, CCL28*
	PSC-IBD_{LT}	5/8 UC, 3/8 CD	FFPE	IHC	VAP-1, CD103, E-cadherin, CXCL12
				ISH	*ITGB7, CCL28, CCL25*
			Frozen	IHC	MAdCAM-1
				qPCR	MAdCAM-1, CCL28, CCL25, CK7
Controls	Control	n.a.	FFPE	IHC	VAP-1, CD103, E-cadherin, CXCL12
				ISH	*ITGB7, CCL28, CCL25*
			Frozen	IHC	MAdCAM-1
				qPCR	*MAdCAM-1, CCL28, CCL25, CK7*
	PBC	n.a.	FFPE	IHC	CD103, E-cadherin, CXCL12
				ISH	*ITGB7, CCL28*
	HCV	n.a.	FFPE	ISH	*ITGB7*

PSC, Primary Sclerosing Cholangitis; IBD, Inflammatory Bowel Disease; ST, short-term; LT, long-term; UC, Ulcerative Colitis; CD, Crohn's disease; PBC, Primary Biliary Cholangitis; HCV, Hepatitis C Virus; FFPE, Formalin-Fixed Paraffin Embedded; IHC, Immunohistochemistry; ISH, In Situ Hybridization

1b. Used colonic tissue

Group	Type	IBD type	Condition	Technique	Target
	PSC-IBD	18/18 UC	FFPE	IHC	MAdCAM-1, CD103, E-cadherin, CXCL12
			Frozen	PCR Array	
	IBD	11/11 UC	FFPE	IHC	MAdCAM-1, CD103, E-cadherin, CXCL12
			Frozen	PCR Array	

PSC, Primary Sclerosing Cholangitis: IBD, Inflammatory Bowel Disease; UC, Ulcerative Colitis; FFPE, Formalin-Fixed Paraffin Embedded; IHC

Supplementary table 2 - Patient characteristics of PSC-IBD and IBD patients from which intestinal tissue was used

	PSC-IBD (n=18)	IBD (n=11)	**P-value**
Male [n (%)]	14 (78)	6 (55)	0.0237
Age (years) [median (IQR)]	45 (30-57)	45 (35-57)	0.808
Small duct PSC [n (%)]	4 (22)	-	-
IBD type [n (%)]			-
Ulcerative Colitis	18 (100)	11 (100)	
Age at PSC diagnosis (years) [median (IQR)]	32 (26-44)	-	-
Age at IBD diagnosis (years) [median (IQR)]	27 (21-40)	29 (21-44)	0.611
Disease duration PSC (years) [median (IQR)]	10 (3-16)	-	-
Disease duration IBD (years) [median (IQR)]	14 (7-27)	15 (6-19)	0.947
Montreal Classification [n (%)]			0.528
E1	1 (6)	1 (9)	
E2	2 (11)	3 (27)	
E3	15 (83)	7 (64)	
Endoscopic inflammation [n (%)]	2 (11)	2 (18)	0.622
Medication use [n (%)]			
5-ASA use	15 (83)	8 (73)	0.646
Ursodeoxycholic acid	16 (90)	0 (0)	<0.001
Steroids	2 (11)	1 (9)	1.000
Anti-TNFα	1 (6)	0 (0)	1.000
Thiopurines	5 (28)	5 (46)	0.432

PSC, Primary Sclerosing Cholangitis: IBD, Inflammatory Bowel Disease; IQR, Interquartile range. P-values were calculated using Fisher's Exact Test for categorical variables and Mann-Whitney U statistic for numerical variables.

Supplementary table 3 – Primer sequences

**Gene**	**Forward sequence**	**Reverse sequence**
MAdCAM-1	QT00017367 (Qiagen)	
VAP-1	CAGCAGCGTTTTCAATCAGA	TGCAGAAAACCAGCTGTCAC
CCL28	AATGCAGCAGAGAGGACTCG	TGACAGCAGCCAAGTCACAA
CK7	ACATCGAGATCGCCACCTAC	GATATTCACGGCTCCCACTC
CCL25	CAAAGCTCCACCACAACACG	ATGGAGCCCAGAAATGAGCC
β-Actin	AGAGCTACGAGCTGCCTGAC	AGCACTGTGTTGGCGTACAG
B2M	CTCGCGCTACTCTCTCTTTCT	TGCTCCACTTTTTCAATTCTCT

Supplementary Figure 1

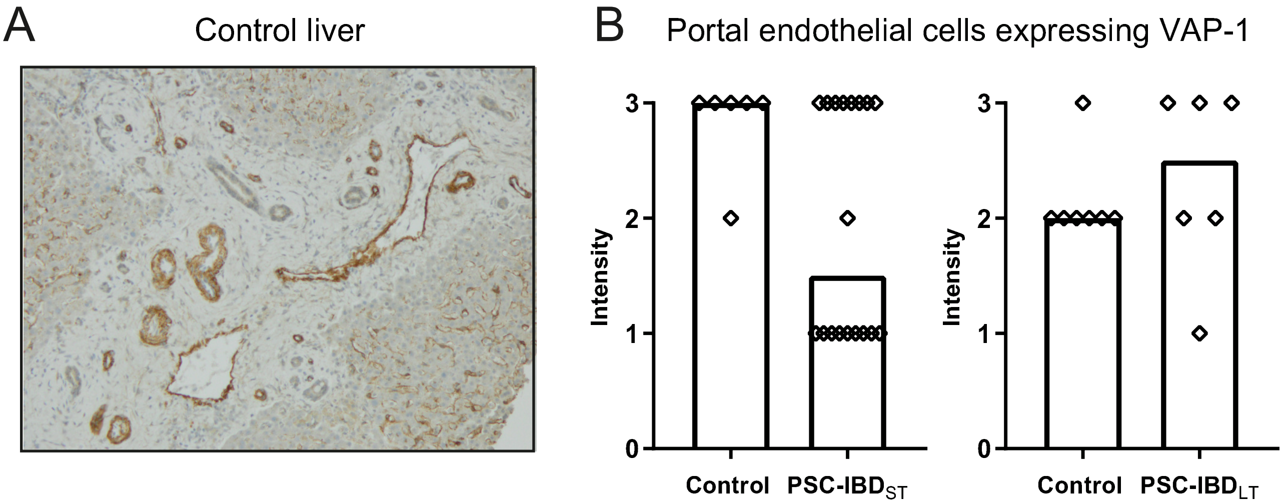

A: Representative picture of immunohistochemistry of vascular adhesion protein-1 (VAP-1) on FFPE liver sections, 10x magnification, control liver. B: Staining intensity (0-3) of VAP-1 on control liver (n=6) and short-term PSC-IBD (PSC-IBD$_{ST}$) liver (n=18) (left) and control liver (n=7) and long-term PSC-IBD (PSC-IBD$_{LT}$) liver (n=6) (right). Statistical testing was performed using the Mann-Whitney U test, data presented as median. A p-value <0.05 was considered statistically significant.

Supplementary Figure 2

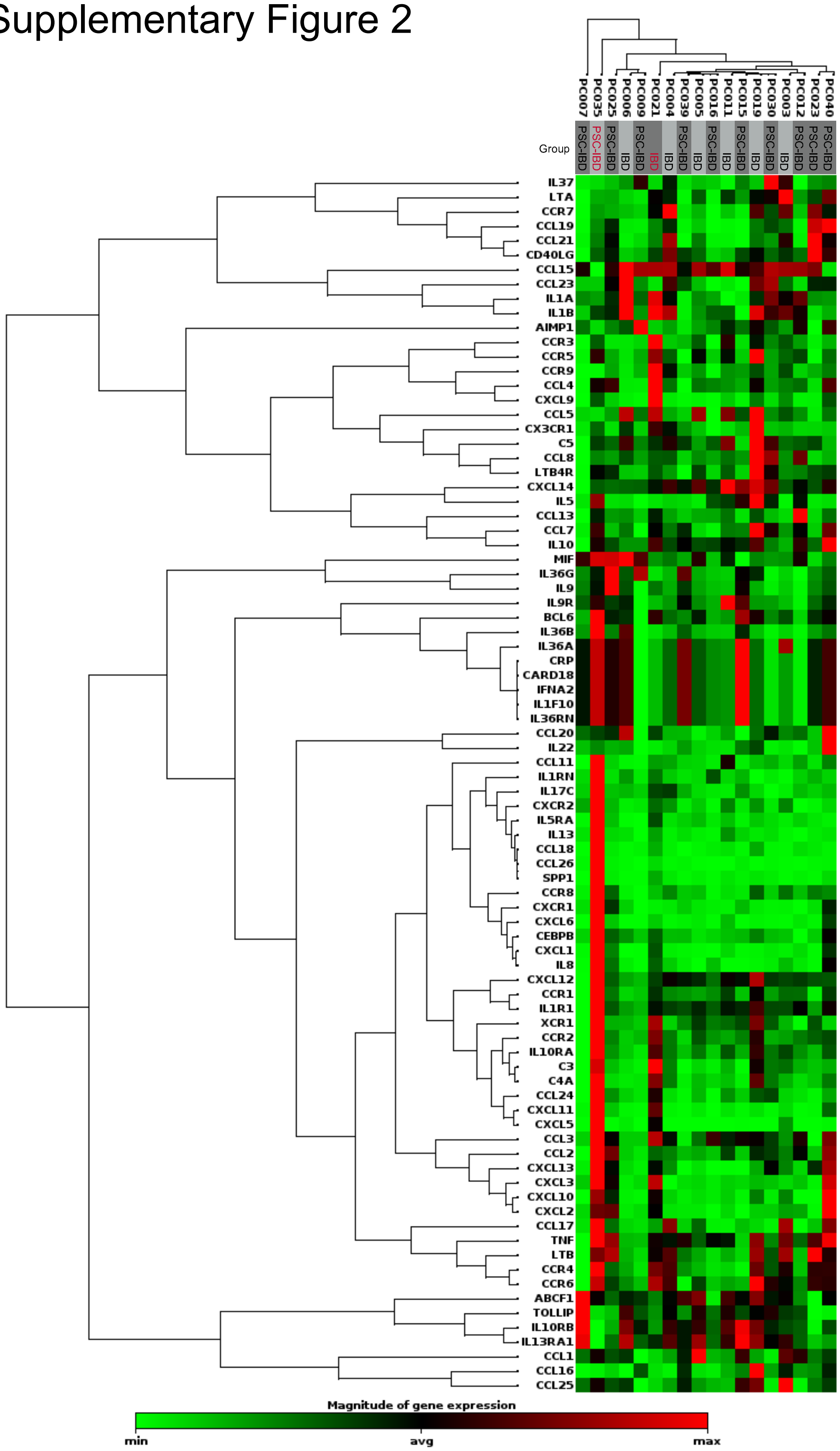

Heat map of gene expression profile of 96 genes encoding for inflammatory cytokines and receptors in colonic biopsies of patients with PSC-IBD (n=11) and IBD (n=7). Samples with endoscopic signs of inflammation are depicted in red.

Supplementary Figure 3

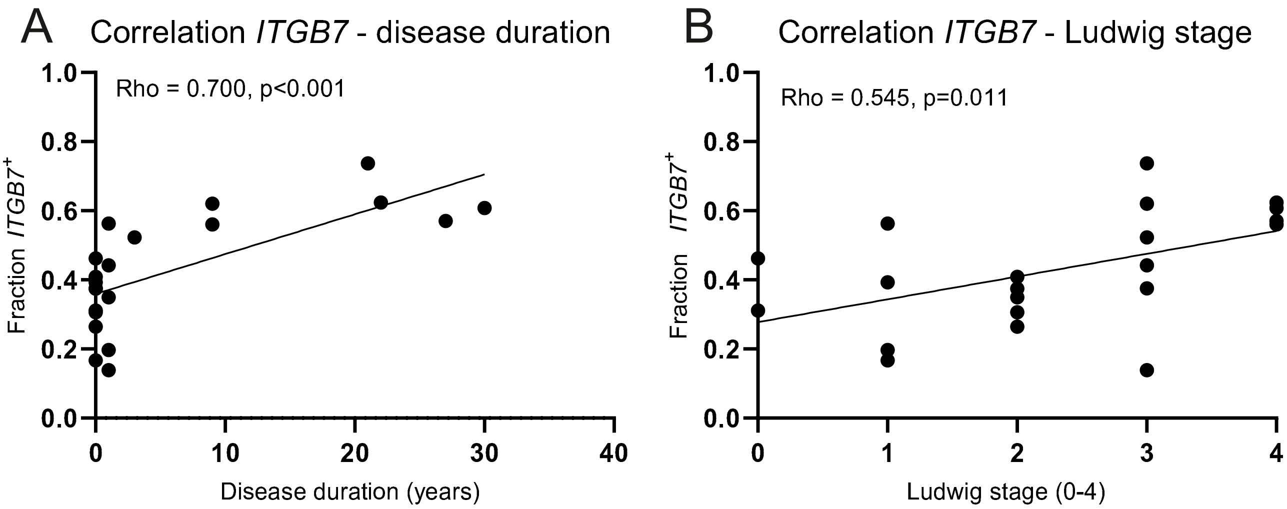

A: Correlation between disease duration in years (PSC-IBD$_{ST}$ and PSC-IBD$_{LT}$ combined) and fraction of CD3+ T-cells that show mRNA expression of ITGB7. B: Correlation between Ludwig stage (PSC-IBD$_{ST}$ and PSC-IBD$_{LT}$ combined) and fraction of CD3$^+$ T-cells that show mRNA expression of ITGB7. Correlation was tested using Spearman's Rho, a p-value <0.05 was considered statistically significant.
